# Benefits of Adding Virginiamycin to *Arapaima gigas* (Schinz, 1822) Diet Cultivated in the Brazilian Amazon

**DOI:** 10.1155/2020/5953720

**Published:** 2020-05-14

**Authors:** Jucilene Cavali, Jerônimo V. Dantas-Filho, Beatriz A. Nóbrega, Lucas Henrique V. Andrade, Rute B. Pontuschka, Paulo Henrique G. Gasparotto, da S. Reginaldo Francisco, Luiz Donizete C. Junior, Marlos O. Porto

**Affiliations:** ^1^Postgraduate Program in Health and Sustainable Animal Production, Federal University of Acre (UFAC), Rio Branco, AC, Brazil; ^2^Postgraduate Program in Environmental Sciences, Federal University of Rondônia, Rolim de Moura, RO, Brazil; ^3^Department of Fisheries Engineering Course, Federal University of Rondônia, Presidente Médici, RO, Brazil; ^4^Postgraduate Program in Animal Biotechnology, Paulista State University, Botucatu, SP, Brazil

## Abstract

The aim of this study was to evaluate the physiological, zootechnical, and environmental benefits of the use of growth-promoting virginiamycin in the pirarucu diet. The research was conducted at the Carlos Eduardo Matiazze Fish Center, Federal University of Rondônia. In this study, 96 pirarucu fish were distributed in excavated nursery, divided into two treatment groups, control (0.0 mg kg^−1^) and treatment (75.0 mg kg^−1^) of feed with virginiamycin, with 8 replications in a completely randomized design. With virginiamycin being incorporated into commercial feed, for 107 days of cultivation, the fish were slaughtered with an average weight of 9.18 kg. Carcass weight, flesh, residues, and internal organs/guts were evaluated to calculate slaughter yields, Spearman's correlation, and subsequently regression. For the quantification of micronuclei, a count of 1,000 cells per blade was determined. The means of the results obtained were contrasted by the Student's *t*-test (*α* = 0.05). Virginiamycin did not cause oscillations in the limnological variables of the nursery but could reduce micronucleated erythrocytes. The average yields in carcass, flesh, and waste were 67.43, 53.4, and 43.14%, respectively. Virginiamycin provided strong correlations (*ρ*^2^) for better productive yields and lower slaughter residue. The hepatosomatic index indicated a strong relationship between liver weight and fat. Virginiamycin may be recommended for fish farming in pirarucu fattening because it contributes to the productive efficiency and sustainability of the fish farm system.

## 1. Introduction

Due to demand of the growing market for quality products, the technology of production systems becomes necessary in order to increase production, economic return of producers, and consumer satisfaction [1]. This increasing of production can expose fish to stressful factors such as high storage densities and constant management [2], causing a drop in immunity, greater susceptibility to diseases, and low water quality [1], causing a drop in immunity and low zootechnical performance [3]. Due to these problems, in addition to adequate productive management, professionals emphasized that the animal nutrition role should seek food and formulations that stimulate the immune system of animals [1].

The importance of the use of additives in aquaculture has been highlighted for its benefits in relation to immunity, food conversion and productivity, and reduction of the mortality rate [4]. Zootechnical additives are used as growth enhancers, as they positively influence the improvement of animal performance and balance of the intestinal microbiota, allowing the reduction of the cost of food without changing the quality [1, 5]. Normative Instruction No. 13 of November 30, 2004, regulates the use of additives intended for animal feed, defining them as any substances or microorganisms intentionally added to food affecting or improving characteristics of food or animal products. They can be classified according to their functions and properties in technological, sensory, nutritional, anticoccidial, and zootechnical additives [6].

Antimicrobials, also known as antibiotics [7], are growth enhancers added to rations that act by decreasing the population of pathogenic microorganisms and the production of toxins by undesirable microorganisms in the digestive tract, minimizing the number of inflammatory cells due to a smaller immune response [5], in addition to the potential to optimize the absorbing capacity of enterocytes [8]. Antibiotics are divided into ionophores and nonionophores, and among the nonionophores available and allowed in the national market is virginiamycin [9] belonging to the class of streptogramins produced by mutant lineage *Streptomyces virginiae*, consisting of two peptides, factor *m* (C_28_H_35_N_3_O_7_) of molecular weight of 525 and factor *S* (C_43_H_49_N_7_O_10_), which have a synergistic effect when combined [10]. Virginiamycin may be responsible for microbial growth control, acts in biochemical processes of protein synthesis, and inhibits stretching of bacteria harmful to gastrointestinal tract health [11].

The state of Rondônia is the largest producer of native fish in Brazil, corresponding to 47.5% of the production of a total of 94,000 tons farmed in the country [12], and has pirarucu as the second most cultivated fish in the north region, which represents about 9% of the fish farmed in Rondônia. Although pirarucu is native, adapted to such hydroclimatic conditions, knowledge about its nutritional requirements is still limited; however, it is known that it is of great social, economic, and environmental importance, being studied in terms of resources fishing grounds and also its use in fish farming [13].

The great challenge for the productive sector and regulatory bodies is to prove to consumers that the use of additives in animal feed, when used responsibly and in accordance with established national standards (Ministry of Agriculture, Livestock and Supply) and worldwide [14], does not leave residues, does not affect water quality, and does not cause risks to fish health, nor to human health. Studies show that virginiamycin is not genotoxic to teleost fish [4, 15]. Virginiamycin has been used for more than 30 years as a growth stimulator in poultry, cattle, and pork production due to its potential yield enhancer [16]. However, its use is still little explored in aquaculture, especially in native species of the Amazon, especially carnivorous habit fish such as pirarucu *Arapaima gigas* (Schinz, 1822).

The objective of this study was to evaluate the physiological, zootechnical, and environmental benefits of the use of growth-promoting virginiamycin in the pirarucu diet (*Arapaima gigas*).

## 2. Materials and Methods

The study was conducted at the Carlos Eduardo Matiazze Fish Farming Center, Federal University of Rondônia, Presidente Médici, RO. Farming/breeding was carried out under the consent of the Ethics Committee on the Use of Animals, under protocol no. 001/2019, in a dug nursery with flow of 5 liters s^−1^, dimension of 1,000 m^2^, depth of 1.64 m, subdivided into 16 hapas with an area of 50 m^2^ each, subdivided into galvanized canvas and coated with vinyl polychloride (PVC), and end of floating feeders of 1.5 m radius.

In this study, we used 96 pirarucu fish with an initial weight of 7.4 ± 0.13 kg distributed in 16 hapas, totaling 6 fishes per hapa, and divided into two treatment groups—control (0.0 mg kg^−1^) and treatment (75.0 mg kg^−1^) with virginiamycin—with 8-hapa replications in a completely randomized design. Commercial virginiamycin (VM 10% Agrofish Agricultural Company Ltd., protocol no. 09176 DCB/ANVISA and no. 98455-68-6—*Chemical Abstract Service Registry*® (CAS) [17]) was diluted in 96% alcohol and incorporated into the ration by daily sprinkler 30 minutes before the supply of feed, as well as the sprinkler only of the alcoholic solution in the feed without the additive for sensory standardization. Extruded commercial feed containing 36% crude protein (Table 1) was provided at a feeding rate of 1% of body weight at 10:00 a.m. and 5:00 p.m. for 107 days.

Limnological parameters evaluated monthly in nurseries were hydrogenic potential (pH), dissolved oxygen (mg L^−1^), conductivity (*μ*s), and temperature (°C) *in situ*, through a previously calibrated multiparametric probe; total ammonia (*μ*g L^−1^) was measured in the laboratory, and transparency of the water of hapas (cm) was determined in situ using the Secchi disc [17–19]. However, in monthly biometrics, individual weighing of animals was performed for adjustments in the trawling rate and calculations of apparent food intake and conversion (CA) and average weight gain (GP) per hapa. To perform the morphometric evaluations, the following variables were measured: CT, total length; CP, standard length; LDC, cranial dorso-width; DC, medium body diameter were performed.

Morphometric and yield correlations were calculated by Spearman's method, and the effects of simple correlation between medium body circumference (PC), total length (CT), standard length (CP), total weight (PT), weight gain (GP), and apparent feed conversion (CA) were obtained. Also, for the means of total weight (PT), viscera (guts) (VC), carcass yield in relation to body weight (RCTPC), manta yield in relation to body weight (RMTPC), manta yield in relation to carcass weight (RMPCA), and yield in slaughter residue (RD) were obtained. Subsequently, regression was performed for the variables that most suffered from the effect of the use of virginiamycin between the correlations performed. Luxinger et al. [11] suggest calculating the variables body circumference, yield on slaughter residue, and total weight, as variable performance indicators. In the final net-fishing the fish were removed individually, and the blood collection of three fish from each hapa was quickly harvested preceding the slaughter, with the aid of a 3 mL heparinized syringe or one containing anticoagulant EDTA (ethylenediamine tetraacetic acid) 10%, following the guidelines of Furnus et al. [20] and Opiyo et al. [19].

Then the slaughter by exsanguination was performed by cutting the gill arches and medullary section followed by bloodletting facilitated in water/ice in the proportion of 2 : 1, in chlorinated water at 5 ppm, followed by storage at −20°C for 10 hours [21]. For processing, the animals were washed and gutted, removing the leather with scales, head by section at the height of the junction with the spine, and viscera after performing a longitudinal cut. In the bone of the carcass, it was possible to quantify the fractions of spine, flesh, and bones. The guts were separated as visceral fat fractions and internal organs (heart, spleen, liver, intestine, and stomach). Using the weight of the animal and the carcass weight it was possible to calculate the carcass yield and correlate it to the weight of the animal before the bleeding to obtain the yields. Carcass yield (RC) = (carcass weight × 100)/total weight; manta yield (MRI) = (weight of the blanket × 100)/total weight; percentage of viscera (PV) = (weight of viscera × 100)/total weight; and fat percentage (PG) = (internal fat in this weight × 100)/total weight.

In the laboratory, slides were made in triplicate with blood smear and stained with panoptic dye following the suggestions of Aly and Albutti [22] and Mattos et al. [4]. Subsequently, micronucleated erythrocytes were quantified by counting 1,000 cells per blade under an optical light microscope as recommended by Aly and Albutti [22]. According to Furnus et al. [20], the micronucleus presence test is a cytogenetic technique used to ascertain the influenced genotoxicity of the cultivation environment. For this, the evaluation is applied to the peripheral blood of fish as recommended by Furnus et al. [20]. Following the propositions prescribed by Aly and Albutti [22], the following criteria for micronucleus identification were met: (1) the absence of links with the main nucleus; (2) the same intensity of the main core color; (3) the size with a ratio of 1/10 to 1/30 of the main core size, and (4) the same focal plane.

With the reduction of the weight of the viscera and also of the liver and due to the little variation in the weight of visceral fat, the hepatosomatic index (HSI) was calculated, which is the correlation of liver weight and visceral fat.

Qualitative data were submitted to the contrast comparison test between the means by Student's *t*-test (*α* = 0.05). To perform the statistical analyses, genes software [23] was used for the necessary calculations and for facilitating the retraction and interpretation of the results.

## 3. Results

In the study period, the water quality parameters of the nursery presented mean values of 4.76 mg L^−1^ of dissolved oxygen, 1.14 *μ*g L^−1^ of total ammonia, 70.68 *μ*s of conductivity, 6.89 of hydrogenic potential, 30.82°C of temperature, and 61.91 cm of transparency.

After 107 days of breeding, the animals obtained an average of 9.18 kg of body weight, an average gain of 2.15 kg in this period. The standard length averaged 96.76 cm and head length averaged 35.0 cm (Table 2).

The virginiamycin additive influenced all carcass yield and pirarucu slaughter residue variables (Table 3). Secondary differences were verified in the variables total weight and blanket (Table 4). It is noteworthy that the additive provided lower weight in viscera with values from 0.46 to 0.41.

The correlation coefficients (*ρ*^2^) were significant, higher than 0.79 (Table 4), according to Spearman's test. It is worth noting that the effects of virginiamycin on the variables are presented, with treatment groups (control 0.0 mg, treatment 75.0 mg) doses of virginiamycin in the rations.

Among the variables measured, the ones that had the most prominent effects were body circumference, carcass yield, flesh/meat yield in relation to body weight, meat yield in relation to carcass weight, weight gain, and apparent feed conversion. Yield on slaughter residue and weight of viscera/guts had less effects (Table 4).

**Figure 1:**
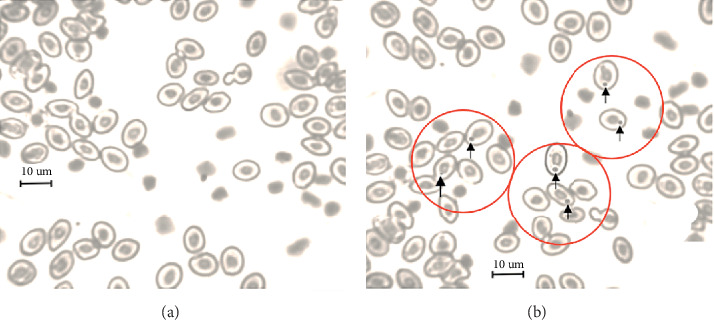
*Arapaima giga* blood cells. (a) Normal erythrocytes. (b) Micronucleated erythrocytes found in the blood circulation of the pirarucu.

In the morphometry of the internal organs and in the number of micronuclei per blade, there were significant differences between treatment and control groups (Table 4). Some erythrocytes presented themselves circularly, with oval nuclei centered on the cytoplasm and easy identification of micronucleus cells (Figure 1).

However, a smaller number of micronucleus blood cells were observed in treatment with 75.0 mg of virginiamycin added to the feed (Table 3). The result demonstrated that the antibiotic used, besides not presenting toxicity, may have contributed to the improvement of the physiological system of animals by decreasing micronucleus erythrocytes (Table 3). According to Tables 2–5, the 75 mg kg^−1^ dose of virginiamycin in the feed did not cause a negative effect (*p* > 0.05) on the body weight of pirarucu; on the contrary, it improved body weight and carcass yields (*p* < 0.05) and decreased residue yield (*p* < 0.05).

**Figure 2:**
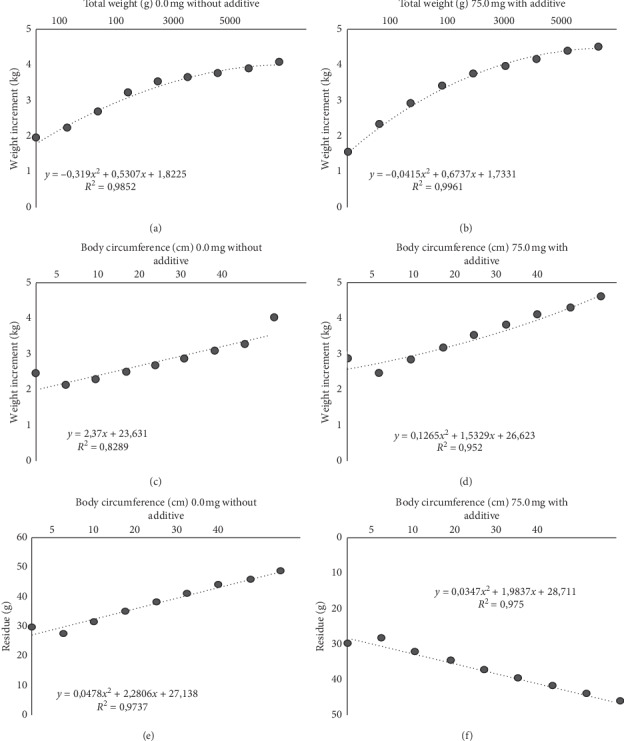
Regression to total weight, body perimeter, and yield of pirarucu residue grown under treatment with and without virginiamycin.

The hepatosomatic index (HSI) was calculated, based on the results summarized in Table 4 and Figure 2. An HSI of *ρ*^2^ = 0.989 was observed, which is a strong relationship between weights, since, as the liver weight decreased, the weight of visceral fat also decreased, including liver fat which may have decreased with the use of virginiamycin.

Regressions were calculated according to the development of the pirarucu, in order to trace the trend lines. And in order to facilitate the visualization and interpretation of the data, averages of total weight, body perimeter, and yield in residue of pirarucu for each hapa of the nursery were presented; therefore, 8 points were set in the graphs (Figure 2), under the effect of 0.0 mg and 75 mg kg^−1^ of the additive virginiamycin.

Concomitantly, with the results presented the dispersion enunciated by nonlinear regression (Figure 2) confirmed that the variables total weight, body perimeter, and yield on slaughter residue had an effect by the use of the antibiotic virginiamycin in pirarucu feed. Therefore, virginiamycin has the potential to improve productive performance and reduce waste yield in the slaughter of pirarucu when added to food (Table 3).

## 4. Discussion

The nursery water quality used remained within the limits recommended for the cultivation of pirarucu according to Drumond et al. [5], Ezike et al. [24], and Okey et al. [25]. Thus, it can be assumed that the physicochemical parameters of the water of the nurseries were adequate to the physiological characteristics of the pirarucu. The inclusion of virginiamycin in the feeding of tropical fish grown in excavated and semiexcavated nurseries has not caused significant fluctuations in limnological variables [8]. In this sense, virginiamycin is not able to cause a negative effect on water quality parameters [20, 26]. Thus, it can be assumed that the physicochemical parameters of the water of the nursery in the weakness of the antibiotic did not disturb the metabolic functioning of the animals [22, 23, 27, 28].

The evaluation of zootechnical performance allows slight detection of food quality and animal well-being in relation to the environment because stress may occur due to discomfort, which imposes susceptibility to diseases [5, 25]. In addition, this information can be used by veterinary inspection to evaluate and control the physiological state of pirarucu, standardizing ideal conditions in feeding, minimizing feed waste in cultivation, and reducing discharge nutrients in the environment [24, 29].

Inadequate management of fish in farming systems, such as poor water quality, causes stress that can compromise adaptive capacity to the environment and even cause a temporary interruption of growth [30]. The application of the studied antimicrobial allows optimization of food management; consequently, with adequate control in food management, excessive input of organic matter is inhibited, due to the balance of the rates of floating solids in water nurseries, nitrogen (N) and phosphorus (P) contents, biochemical oxygen demand, potential oxidoreduction, and plankton biodiversity [28].

Optimization of the apparent feed conversion index and weight gain was observed, in addition to increase in carcass, meat, and organ yields [22]. While the reduction in liver weight occurs after food intake, this organ stores carbohydrates and lipids, being considered an initial source of endogenous energy for fish [30], and variations in the amount of fat and/or glycogen stored in the liver significantly influence the weight of this organ as had been verified for years by Franco et al. [31]. The lower liver weight in the treatment with the addition of virginiamycin, from 72.50 to 65.90 g, may have occurred due to a synergism between the additive and cholic acid causing reduction of liver fat, naturally present in this organ according to Figueiredo et al. [32].

Martorell et al. [33] and Dhama et al. [34] tried *Oreochromis niloticus* and also found a strong relationship between liver weight and visceral fat, *ρ*^2^ = 0.966 ± 0.03, indicating that with the use of virginiamycin there may be reduction of fat in the liver, as verified in this study. A lower accumulation of cholesterol in the hepatic wall is favored by cholic acid in the conversion of bile acids, potentiating the effect of dietary cholesterol, increasing its absorption, and not accumulating lipids in the liver, avoiding the so-called fatty liver in animals as discussed by Figueiredo et al. [32]. The efficacy of the use of morphometric ratios in pirarucu was confirmed in the study presented here, because the majority of the coefficients of determination were significant, and they were higher than 0.79 for pirarucu weights. Reis-Neto et al. [35] reported that body measurements were effective in estimating body growth. Reis-Neto et al. [35] also researched *Brycon orbignyanus* and suggested that the body perimeter taken in the insertion of dorsal fin is adequate to determine weight increment and total weight of species.

The direct effect of body perimeter, weight with carcass, total weight, meat yields, weight gain, and feed conversion on correlation observed here was also reported by Martorell et al. [33], who studied pirarucu morphometry and observed a linear relationship between length and total weight, with equations presenting coefficients of determination above 0.96. Diodatti et al. [36], in a study conducted with tilapia (*Oreochromis niloticus),* observed a significant correlation coefficient between carcass yield and body perimeter. Divergences of morphometric measurements correlated to the weight of some parts of the body may occur, since each species presents specific variations in body shape, so it is recommended that some variables calculated in the Spearman's correlation be replicated [31, 36].

The weights of viscera, leather, and spine/bone are classified by agribusiness as slaughter residues, so that the lower the weight, the higher the profitability per animal [33]. Meat yields, weight gain, and body circumference also showed a strong correlation with carcass weight in the study by Luxinger et al. [11]. In general, the measurements and morphometric ratios of pirarucu were directly related to total body weight and body components [37]. However, studies related to this species are scarce in the literature, so future research into the evaluation of phenotypic correlations in pirarucu should be performed. However, providing virginiamycin to fish promotes an increase in most yields, including carcass yields; the exceptions are residues and fat [34].

The importance of the use of the additive studied in fish farming stands out for its benefits, such as improving immunity, achieving physiological balance, increasing productivity, and invigorating intestinal-absorbing functioning, in addition to improving food efficiency and reducing the mortality rate [4]. Because when fish consume plankton and/or even feed, bacteria normally present in the intestine use these foods to multiply; in this process energy, proteins, vitamins and gases are produced [22]. These bacteria are divided into two groups, Gram-positive and Gram-negative. Gram-negative group bacteria are considered excellent for the organic development of the digestive system because they produce propionic acid that is a precursor to energy for animals [38]. For this, virginiamycin acts by controlling the growth of Gram-positive bacteria, which is advantageous to the animal, because Gram-positive bacteria compete for food without generating benefits to the body [22]. With the growth of Gram-negative bacteria controlling Gram-positive ones, there are more nutrients available; Gram-negative bacteria grow in greater quantity, predominantly on Gram-positive bacteria, which ensures better use of food and inhibits the invasion of pathogenic microorganisms [8, 34].

Because of this, it can be understood that the dose of virginiamycin offered was favorable for pirarucu physiology, invigorating the absorbing functions of intestinal cells [4]. Knowledge and monitoring of fish physiology have been an important tool in intensive breeding of several species of teleost fish [29, 39], and results obtained were useful in improving the physiology of pirarucu, making it more resistant to the more dense lysed cultivation model. Fish biomonitoring is important to indicate the quality and sustainability of the growing environment [40]. The pollution of the aquatic environment measured as a stress biomarker comprises a wide range of parameters, such as hematological, physiological, and genotoxic ones [39].

Micronuclei are indicators of stress and environmental conditions; these inflammatory cells arise from whole or fragmented acentric chromosomes that are postponed during anaphase, due to the lack of pairing chromosomes and/or damage to the mycotic spindle [41]. This damage occurs due to adverse environmental conditions in running water pollution effect [42]. Anthropogenic action pollutes groundwater and water from streams and springs, launching genotoxic compounds in fish farms, causing uncomfortable conditions to fish [20, 39].

The data presented in Table 3 show lower number of micronuclei, from 26.33 to 23.59, indicating that virginiamycin may have contributed to the physiological system of pirarucu. Another study conducted by Pontuschka and Hurtado [43] with *Colossoma macropomum* showed probable exposure of fish to substances and/or environmental conditions of genotoxic potential, so that the micronucleus test and weight-length ratio presented correlation that may be useful for biomonitoring of contaminated environments. Therefore, undoubtedly micronuclei indicate stress to adverse environmental conditions, such as low water quality and high storage intensity, weakening the fish immune system [29]. Thus, virginiamycin allows fish to bypass the effects of environmental stress, because even in adverse environment this additive can contribute to the best use of available food [16].

The frequency of micronuclei observed at a given time can be considered a complex response between genotoxic activity and the efficiency of the body's physiological defense mechanism [27], with a higher incidence of micronuclei after exposure of the organism to different pollutants as well as natural conditions or through exposure in a controlled environment [22]. Micronuclei detected in the treatment do not show virginiamycin as toxic potential [8, 44]. Examples include the results found by Silva and Nepomuceno [45] in *Pimelodus maculatus* (2.5%), by Rocha et al. [46] in *Colossoma macropomum* (2.4%), and by Grisolia et al. [42] in several fish system species in South America (<1.86%), and these frequencies were considered basal levels of the species.

Adverse environmental conditions also affect erythrocytes levels and Na^+^ activity. K^+^ and ATPase, which can be reduced due to environmental stress, negatively influence osmotic balance and amino acid transport, enthalpy of the bloodstream, and the functional stability of vital organs. These factors cause the apathy of metabolism and protein synthesis [47]. The immune system of fish can be divided into innate and adaptive immunity, and the adaptive system is triggered in cultivation stress and indicated by micronucleated erythrocytes [30]. The innate immune system has several active proteins, such as lysozyme and lectins. Lysozyme acts by attacking and destroying the cell wall polysaccharides of different bacterial species, killing microorganisms. Key events in innate immune defense include the recognition of microbial targets for lectins, such as collectins [48]. These proteins recognize foreign cells as “unowned” by carbohydrates expressed on the surface acting as opsonin and stimulating their destruction by phagocytic cells [49].

Fish differ from mammals and birds in relation to acquired immune response, so lectins and other innate immune agents can have much more important roles. Several fish lectins are believed to be aware of pathogens in the immune system [48]. Lectin stimulated by zootechnical additives was isolated in *Rachycentron canadum* ovaries, presented antibacterial activity against *Escherichia coli*, and also reduced the frequency of micronucleated erythrocytes [30]. According to Santos et al. [28], erythrocytes present a high incidence of micronuclei after exposure to different pollutants or pollution agents, either in natural conditions or through exposure in a controlled environment.

The physiological, hematological, and immune conditions of teleost fish are directly related to well-being in the medium culture and correlated with the deposition of muscle proteins, because they are correlated with yield and mass gain [50]. When the conditions of cultivation are favorable, that is, when there are biochemical and physiological stability, there is greater deposition of intramuscular proteins, because cell membranes release fat raising concentrations of free fatty acids in the fluids and are converted into acetyl-CoA, which is used as an energy source, which saves proteins. Thus, energy is directed to somatic, muscular, and bone growth [51].

Studies on stress conditions pointed to greater direction of energy obtained from food to maintain physiological balance and, in a smaller amount, somatic growth [2, 25, 41]. This may justify the significant differences in income between the control and the treatment group. In order to prove this, the apparent feed conversion index is used as an indicator of the quality and supply of the diet [25], as it represents the efficiency of the conversion of food into biomass [28].

Some monitoring studies reported that, in fish farms surrounded by urban areas, fish presented numbers of micronucleated erythrocytes above the tolerated number, with cases exceeding five times the recommended number [42, 52]. The components present in discharges of household and agricultural and industrial waste can cause biochemical and genotoxic changes in teleost fish [42], such as pirarucu. Therefore, micronucleated erythrocytes indicate that nurseries are contaminated by pollutants of genotoxic origin, such as domestic sewage [52] and/or agricultural pesticides [42].

Another aggravating factor is that even fish farms far from areas of urban pollution, if they do not have sedimentation nursery, also called a purification tank, are susceptible to the same environmental and physiological problems, as reported by Bujjamma et al. [8]. These polluting agents are metals such as mercury and iron, pharmaceuticals, polycyclic aromatic hydrocarbons, and contaminated or inadequately degraded fertilizers [53]. It is known that the costs of feeding in pirarucu farming are too high, because fish are carnivores and require rations with high crude protein levels [54]. However, when offering virginiamycin as an additive in food, there is efficiency in food use and zootechnical performance [27]. By reducing environmental pollution by inhibiting feed waste, food costs can be reduced [54]. In addition, Dhama et al. [34] found that the damage from the dissipation of feed in supply to fish within 30 days is sufficient to recruit two employees for the property. However, in the correct incorporation of virginiamycin and in good cultivation conditions, the period of preslaughter weighting can be shortened [29].

In extension, virginiamycin maximizes feed consistency, minimizes organoleptic losses, and extends life in storage [8]. In a fish farming the most important economic factor is feed, 85% of production costs [27]. Thus, it is necessary to include a liaison agent to ensure proper feeding of the fish [38]. Virginiamycin has safety potential, allowing its use as a chemotherapeutic agent for the treatment of infectious diseases at environmental stress outflow [22]. Despite being a relatively costly additive to rural producers, currently costing 40 to 50 reais (Brazilian currency) per kilo, it can reduce production costs, providing expansion of the productive area and job creation, which promotes the enhancement of working capital of the fish farming [22].

## 5. Conclusions

In conclusion, virginiamycin added to the ration provided pirarucu with lower weight of viscera/gut and weight of internal organs such as the liver due to lower fat deposition. It provided better morphometric correlations of yield and decrease of slaughter residue. Likewise, it reduced micronucleated erythrocytes. The antibiotic can be recommended for fish farming in pirarucu fattening because it contributes to the productive efficiency and sustainability of the cultivation system. As know-how, evaluations of the use of the additive are suggested in the younger phases of pirarucu and under longer administration for carnivorous species, as well as via other methods or vehicles including the additive.

## Figures and Tables

**Table 1:** Levels of ration guarantee offered for pirarucu grown in Western Amazonia.

Composition	Content (g kg^−1^)	Composition	Content (g kg^−1^)
Dry matter (g)	910.0	Ethereal extract (min, g)	80.0
Crude protein (min., g)	360.0	Calcium (max., g)	35.0
Fibrous matter (max., g)	95.0	Calcium (min., g)	20.0
Mineral matter (max., g)^1^	15.0	Phosphorus (min., g)	15.0

**Table 2:** Average of morphometric and zootechnical performance of pirarucu fed with diets with and without adding antibiotic virginiamycin.

Variables	Treatment groups		*p* value	CV (%)
	Control	Treatment		
Total weight (kg)	9.18	9.97	0.044	2.97
Overall length (cm)	104.6	104.0	0.001	1.58
Standard length (cm)	96.81	96.70	0.003	1.85
Head length (cm)	40.00	30.00	0.017	12.25
Average body circumference (cm)	41.30	45.60	0.029	22.05
Apparent feed conversion	3.75	3.21	0.032	31.85
Total weight gain (kg)	1.60	1.77	0.036	17.75
Daily weight gain (g day^−1^)	16.16	17.09	0.030	17.79

The average result of the body perimeter was better with the additive, from 41.3 to 45.6 cm (*p* < 0.05), although another important indicator is the apparent feed conversion despite having had an ineffective mean of 3.4 (Table 2).

**Table 3:** Average carcass yield, slaughter residue, weight, and the number of micronuclei present in the blood circulation of pirarucu fed with ration administered under different doses of the antibiotic virginiamycin.

Variables	Treatment groups		*p* value	CV (%)
	Control	Treatment		
Total weight (kg)	8.78	8.55	0.044	5.42
Leather and scales (kg)	1.45	1.40	0.001	5.83
Head (kg)	0.98	0.95	0.032	5.34
Total offal (kg)	0.46	0.41	0.036	8.24
Carcass (kg)	5.89	5.71	0.030	4.88
Backbone (kg)	1.22	1.21	0.017	9.82
Fish blanket (kg)	4.71	4.67	0.047	9.78
Visceral fat (kg)	0.123	0.119	0.061	16.4
Carcass yield in relation to body weight (%)	67.12	67.73	0.001	1.11
Total body weight yield (%)	52.69	54.17	0.001	4.63
Total weight of carcass (%)	78.09	79.83	0.016	4.31
Residue (kg)	4.29	3.98	0.033	10.58
Slaughter residue yield (%)	47.91	46.36	0.001	5.84
Liver (g)	72.50	65.90	0.032	6.44
Heart (g)	9.75	9.83	0.001	14.47
Stomach (g)	64.17	62.92	0.001	7.08
Spleen (g)	3.25	3.38	0.016	22.22
Intestine (g)	80.33	80.50	0.033	17.10
Micronuclei per blade	26.33	23.59	0.001	2.90

**Table 4:** Spearman's correlation coefficients of morphometric and yield variables of pirarucu fed (control 0.0 mg, treatment 75.0 mg) with virginiamycin antibiotic.

Variables	PC	CT	CP	PT	GP	CA
PC	—	+0.456/0.493	+0.472/0.483	+0.419/0.806	+0.421/0.636	+0.444/0.646
CT	+0.426/0.493	—	+0.409/0.424	+0.924/0.917	+0.412/0.404	+0.442/0.404
CP	+0.472/0.483	+0.409/0.424	—	+0.699/0.734	+0.419/0.433	+0.319/0.403
PF	+0.419/0.806	+0.924/0.917	+0.699/0.734	—	+0.599/0.894	+0.629/0.791
GP	+0.421/0.636	+0.412/0.404	+0.419/0.433	+0.599/0.894	—	+0.798/0.899
CA	+0.444/0.646	+0.442/0.404	+0.313/0.303	+0.629/0.791	+0.798/0.899	—

*ρ* ^2^: 0,877. PC: mean body circumference, TC: total length, CP: standard length, PT: total weight, GP: weight gain, and CA: apparent feed conversion.

**Table 5:** Spearman's correlation coefficients of morphometric variables in ratio of total weight and productive performance of pirarucu (control 0.0 mg/treatment 75.0 mg) of the antibiotic virginiamycin.

Variables	PT	PC	VC	RCTPC	RMTPC	RMPCA	RD
PT	—	+0.690/0.960	+0.512/0.516	+0.612/0.606	+0.507/0.713	+0.490/0.507	+0.612/0.406
PC	+0.712/0.906	—	+0.399/0.336	+0.492/0.790	+0.577/0.799	+0.750/0.409	+0.551/0.802
VC	+0.512/0.516	+0.399/0.336	—	−0.577/0.608	−0.489/0.579	−0.516/0.695	+0.812/0.506
RCTPC	+0.612/0.606	+0.492/0.790	−0.577/0.608	—	−0.380/0.379	−0.380/0.379	−0.516/0.695
RMTPC	+0.507/0.713	+0.577/0.799	−0.489/0.579	−0.380/0.379	—	+0.412/0.506	−0.610/0.797
RMPCA	+0.490/0.507	+0.551/0.802	−0.516/0.695	−0.380/0.379	+0.412/0.506	—	−0.511/0.799
RD	+0.612/0.406	+0.750/0.409	+0.812/0.506	−0.516/0.695	−0.610/0.797	−0.511/0.799	—

*ρ* ^2^: 0,799. PC: mean body circumference, TC: total length, FP: final weight, VC: viscera, RCTPC: ratio of carcass yield to body weight, RMTPC: ratio of manta yield to yield body weight, RMPCA: ratio of manta yield to carcass weight, and RD: slaughter residue yield.

## Data Availability

The data used to support the findings of this study are available from the corresponding author upon request.
